# Can we interrogate public databases to fill critical gaps in mental health epidemiology? Testing the association between cannabis and psychosis in the UK as an example

**DOI:** 10.1017/S2045796023000537

**Published:** 2023-06-15

**Authors:** Gianfranco Di Gennaro, Marco Colizzi

**Affiliations:** 1 Department of Health Sciences, Magna Graecia University of Catanzaro, Catanzaro, Calabria, Italy; 2 Department of Medicine, University of Udine, Udine, Friuli-Venezia Giulia, Italy

**Keywords:** epidemiology, health outcomes, schizophrenia, social environment, statistics

## Abstract

The psychoactive properties of cannabis have been known forever. Since 1987, several prospective studies have suggested an increased risk of psychosis among cannabis users, with alternative explanations failing to account for such an effect. A cause–effect relationship has thus been implied. Further evidence has indicated that there is a dose–response relationship, and high-potency cannabis varieties confer the greatest risk of psychosis. As cannabis use has become more common over the last decades, one would expect a related increase in the number of schizophrenia cases. However, evidence in this regard remains equivocal for several reasons, including relying on databases that are not primarily designed to address such question and the issue that solid information regarding the incidence of schizophrenia is a relatively recent acquisition. Recent years have seen the development of online web publications, such as Google Trends and “Our World in Data”, where data are explorable and interactable for tracking and comparing trends over specific periods and world regions. By using such databases, we believe that the question whether changes in cannabis use are associated with changes in schizophrenia rates can be answered, at least partly. Therefore, we tested these tools by evaluating trends in cannabis use and both cases and prevalence of schizophrenia in the United Kingdom, one of the countries where the incident rates for psychotic disorder have been suggested to be particularly increased by cannabis consumption. Crossing data from these tools revealed that interest in cannabis has been growing at the country level for over 10 years, with a parallel overlapping raise in psychosis cases and prevalence. Following up on this example, let us think of how many public health opportunities these public resources may offer. The question now is whether public health interventions for the benefit of the general population will follow suit.

The psychoactive properties of cannabis use have been orally handed down since 2700 BC (Brand and Zhao, 2017), first paid attention by the physician Iban Beitar (1197–1248 BC) (Dhunjibhoy, 1930) and much later brought to the limelight of modern medicine by the French psychiatrist Jacque-Joseph Moreau in 1845 (Moreau, 1845). The latter highlighted how hashish, the cannabis plant–derived resin, could precipitate acute psychotic reactions like those observed in psychosis patients, fuelling the debate up to the current century whether it may have a causal role in the pathophysiology of schizophrenia-spectrum disorders (Abel, 2005). Already in 1930, drug-induced psychosis was considered the most common type of psychosis in India after both affective and non-affective psychoses, with hemp, a cannabis variety, as the main drug suggested (Dhunjibhoy, 1930).

Over the last 20 years, the topic has been set on fire, with reviews suggesting a higher risk of any psychotic outcome in people who have ever used cannabis (Moore *et al.*, 2007), which increases depending on level of use (Marconi *et al.*, 2016), independently of the confounding effect of temporary intoxication (Moore *et al.*, 2007). A dose-dependent effect of cannabis use has been also implicated in psychosis relapse, with increased frequency of use and cannabis potency putting patients at higher risk (Schoeler *et al.*, 2016). Such evidence has raised public health concern, especially considering the increasing consumption of cannabis for recreational purposes over the years, with about 200 million users worldwide. Also, trends in decriminalizing or legalizing cannabis use in many countries are likely to have a further impact on the phenomenon of cannabis-induced psychosis (Colizzi and Murray, 2018).

To establish an association between cannabis use and psychosis, and possibly a cause–effect relationship, the topic has been studied in different lines of research, with converging results (Colizzi and Bhattacharyya, 2020). Several prospective studies have been carried out since 1987, with a follow-up of 1 to 35 years, supporting a twofold increased risk of psychosis as a function of cannabis use (Colizzi and Bhattacharyya, 2020). As alternative explanations, some studies have addressed the confounding effect of other substances (Arseneault *et al.*, 2002) and tobacco (Di Forti *et al.*, 2015), suggesting a specific effect of cannabis in increasing risk of psychosis. Other studies have attempted to control for the confounding effect of preexisting psychopathology in cannabis users, finding that it may reduce (Henquet *et al.*, 2005) or be irrelevant (Arseneault *et al.*, 2002) but not deny the risk of developing psychosis among cannabis users. Further, a self-medication hypothesis has never been established (Spalletta *et al.*, 2007), and although a bidirectional relationship between cannabis use and psychosis may exist (Ferdinand *et al.*, 2005), it is unlikely that there is a full reverse causality entirely accounting for the effect of cannabis on psychosis (Colizzi and Bhattacharyya, 2020). Finally, cannabis use and psychosis have been found to share common genetic susceptibility to a small extent, without per se excluding the possibility that cannabis had a causal role in the development of psychosis (Power *et al.*, 2014).

More specifically, looking at a cause–effect relationship, studies indicate a variation in the incidence of psychosis across Europe (Di Forti *et al.*, 2019), as well as a higher magnitude of the association as a function of cannabis consumption (Di Forti *et al.*, 2015). Consistency of findings also emerges in terms of poorer psychosis outcome (Colizzi *et al.*, 2016) and cognitive function (Colizzi and Bhattacharyya, 2017) in the context of cannabis use. Specificity (Niemi-Pynttari *et al.*, 2013) and temporal sequence (Murray *et al.*, 2017) of the association have been proven along with evidence for a biological gradient (i.e., dose–response association) (Marconi *et al.*, 2016) and coherence from experimental proof that exposure to cannabis main ingredient, delta-9-tetrahydrocannabinol, produces psychosis-related behavioural effects (Colizzi *et al.*, 2019b) that are biologically plausible (Colizzi *et al.*, 2019a).

Of the above, there is an issue around the evidence that cannabis use may play a role in increasing the risk of psychosis that perhaps has dominated the debate more than all other aspects combined. As cannabis use has become more common over the last decades, one would expect a related increase in the number of schizophrenia cases. A study exploring the incidence of schizophrenia in South London reported that it doubled between the sixties and the nineties, with a parallel increase in the rate of cannabis use in the year before symptom presentation for those presenting with schizophrenia (Boydell *et al.*, 2006). However, another study from the UK failed to provide evidence of an increase in the incidence of psychotic disorders because of an increase in cannabis consumption at the population level (Frisher *et al.*, 2009). Evidence in this regard remains equivocal for several reasons, including, but not limited to, relying on databases that are not primarily designed to address such questions as well as the issue that, until about 25 years ago, only a few studies had provided solid evidence regarding the incidence of schizophrenia. In 2004, a large systematic review of 158 studies from 33 countries found substantial variations in the incidence of schizophrenia because of several risk factors, such as gender, urbanicity, and immigration status (McGrath *et al.*, 2004). The fact is that the incidence of schizophrenia is higher in countries such as the UK where high-potency cannabis is very popular in the market compared with countries such as Italy where more traditional and less potent cannabis varieties are still consumed (Di Forti *et al.*, 2019). Anyhow, lacking high-quality, comprehensive studies looking at temporal trends in the incidence of psychosis, especially in the context of cannabis use, we are currently unable to either support or disregard the possibility that a population-level increase in cannabis use has led to an increase in the incidence of psychosis worldwide.

To understand the evolution of a public health phenomenon, recent years have seen the development of online web publications where data are explorable and interactable for tracking and comparing trends over specific periods and world regions (Mathieu *et al.*, 2021). By using such databases, we believe that the question whether changes in cannabis use are associated with changes in schizophrenia rates can be answered, at least partly. Therefore, we tested these tools by evaluating trends in cannabis use and both cases and prevalence of schizophrenia in the UK, one of the countries where the incident rates for psychotic disorder have been suggested to be particularly increased by cannabis consumption (Di Forti *et al.*, 2019). An estimate of the number of cases and prevalence of schizophrenia in the UK was obtained from the so-called “Global Burden of Disease”, a research program that involves thousands of researchers across the globe and looks at mortality and disability related to disease, injury, and risk factors. The results of this project are freely available on the dedicated website (https://www.healthdata.org/gbd) as well as on other providers such as “Our World in Data” (https://ourworldindata.org). Then, the volume of web searches in the UK related to the topic “Marijuana” in the time period 2005–2019 was extracted from Google Trends (https://trends.google.com/), an open-source website that can be used to detect the number of searches that take place over a specific period of time (e.g. daily, weekly or monthly) and over a specific period of interest. It calculates the historical values as the Relative Search Volume (RSV), which is a standardized measure consisting in the percentage of the highest value in the series (Nuti *et al.*, 2014). Google Trends represents one of the most used tools in digital epidemiological studies, and over the past decade, Google searches of specific keywords have been used as indicators of the prevalence of a variety of diseases. Also, they have been used more generally as a tool to identify the level of people’s interest in a health topic (Breyer *et al.*, 2011; Flanagan *et al.*, 2021; Johnson and Mehta, 2014; Schootman *et al.*, 2015; Vasconcellos-Silva *et al.*, 2017). It is clear that this tool is invaluable for both monitoring a population’s behaviour or interests in the past, as well as for making future estimates in order to intervene appropriately through the necessary public health interventions (Dugas *et al.*, 2013; Prasanth *et al.*, 2021). Subsequently, the mean annual RSV of marijuana was calculated for comparison with the number of annual cases as well as the prevalence of schizophrenia over the same period. Finally, to facilitate the graphical comparison, data were represented on the *z*-scores scale. Data analysis supported a strong correlation of the annual mean RSV pattern of cannabis use, with the trajectory of both schizophrenia cases and prevalence in the UK (Figure 1A and B).

**Figure 1.**
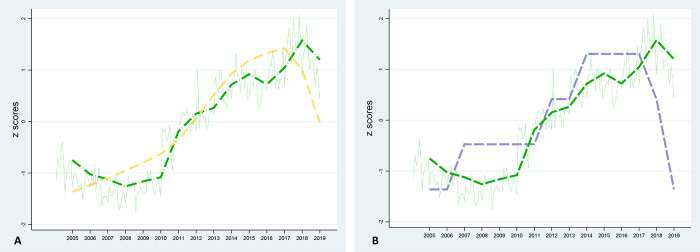
Correlation of the online search volume trajectory of the topic “Marijuana” (green dashed line) with number of cases (orange dashed line (A), Pearson’s *r* 0.87, *p* < 0.001) and prevalence (purple dashed line (B), Pearson’s *r* 0.60, *p* = 0.019) of schizophrenia. Data are expressed as *z*-scores to facilitate graphical comparison. Time span: 2015–2019.

Governments and public health officials need to develop the most effective strategies to tackle the risk of cannabis-induced psychosis in a way that minimizes morbidity and healthcare costs from the potential development of the disorder. The results that we present here are an example of how crossing public datasets could support it. On the one hand, Google Trends tool allows analysts to track interest in cannabis over time in a specific country. Combined with epidemiological data, it serves as input for stakeholders to understand how such interest in cannabis translates in cases of psychosis, with the goal of prioritizing strategies at the country level. Communicating such evidence on the risk of cannabis use is in turn essential for building public trust and reducing the harmful potential of its use.

Establishing a cause–effect relationship between two observed trends is beyond the scope of these resources. Thus, the evidence provided here that interest into cannabis and psychosis rates have increased in a very similar way is not sufficient per se to imply causation. Also, testing these tools revealed another reason for caution in data interpretation, that is the shortness of the time series that makes the presented statistical analysis highly exploratory. However, it is conceivable that such tools can be refined to offer a more systematic study of a specific phenomenon, that is, in the example provided here, a more frequent monitoring of schizophrenia cases and prevalence in order to bring out the volume of online interest in marijuana as a practical info-surveillance and forecasting tool. If we want to understand more about exposure to a risk factor, then we need to know how many people are affected by it. Coming back to our study example, knowing how many people interested in consuming cannabis are out there would allow having our ear to the ground in terms of policy responses or risk perceptions. This dataset fills this gap. It clearly shows that interest in cannabis has been growing at the country level for more than a decade. On parallel, an overlapping raise in psychosis cases and prevalence has been documented over the same period and region. Let us think of how many public health opportunities these public resources may offer. The question now is whether their global rollout will produce evidence that is matched with public health interventions that can be administered quickly and equitably across the country, for the benefit of the general population.

## Data Availability

Data used in this Editorial are freely accessible on the dedicated websites (https://www.healthdata.org/gbd, https://ourworldindata.org and https://trends.google.com/).

## References

[ref1] Abel EL (2005) Jacques Joseph Moreau (1804–1884). *American Journal of Psychiatry* 162(3), 458. 10.1176/appi.ajp.162.3.458 15741461

[ref2] Arseneault L, Cannon M, Poulton R, Murray R, Caspi A and Moffitt TE (2002) Cannabis use in adolescence and risk for adult psychosis: Longitudinal prospective study. *BMJ* 325(7374), 1212–1213. 12446537 10.1136/bmj.325.7374.1212 PMC135493

[ref3] Boydell J, van Os J, Caspi A, Kennedy N, Giouroukou E, Fearon P, Farrell M and Murray RM (2006) Trends in cannabis use prior to first presentation with schizophrenia, in South-East London between 1965 and 1999. *Psychological Medicine* 36(10), 1441–1446. 16854250 10.1017/S0033291706008440

[ref4] Brand EJ and Zhao Z (2017) Cannabis in Chinese Medicine: Are some traditional indications referenced in ancient literature related to Cannabinoids? *Frontiers in Pharmacology* 8, 108. 10.3389/fphar.2017.00108 PMC5345167 28344554

[ref5] Breyer BN, Sen S, Aaronson DS, Stoller ML, Erickson BA and Eisenberg ML (2011) Use of Google Insights for Search to track seasonal and geographic kidney stone incidence in the United States. *Urology* 78(2), 267–271. 21459414 10.1016/j.urology.2011.01.010 PMC4551459

[ref6] Colizzi M and Bhattacharyya S (2017) Does cannabis composition matter? Differential effects of delta-9-tetrahydrocannabinol and cannabidiol on human cognition. *Current Addiction Reports* 4(2), 62–74. 28580227 10.1007/s40429-017-0142-2 PMC5435777

[ref7] Colizzi M and Bhattacharyya S (2020) Is there sufficient evidence that cannabis use is a risk factor for psychosis? In Thompson AD and Broome MR (eds), *Risk Factors for Psychosis: Paradigms, Mechanisms, and Prevention*. Nikki Levy, 305–331.

[ref8] Colizzi M, Carra E, Fraietta S, Lally J, Quattrone D, Bonaccorso S, Mondelli V, Ajnakina O, Dazzan P, Trotta A, Sideli L, Kolliakou A, Gaughran F, Khondoker M, David AS, Murray RM, MacCabe JH and Di Forti M (2016) Substance use, medication adherence and outcome one year following a first episode of psychosis. *Schizophrenia Research* 170(2–3), 311–317. 26718334 10.1016/j.schres.2015.11.016

[ref9] Colizzi M and Murray R (2018) Cannabis and psychosis: What do we know and what should we do? *British Journal of Psychiatry* 212(4), 195–196. 10.1192/bjp.2018.1 29557759

[ref10] Colizzi M, Weltens N, McGuire P, Lythgoe D, Williams S, Van Oudenhove L and Bhattacharyya S (2019a) Delta-9-tetrahydrocannabinol increases striatal glutamate levels in healthy individuals: Implications for psychosis. *Molecular Psychiatry* 25(12), 3231–3240. 30770892 10.1038/s41380-019-0374-8 PMC7714685

[ref11] Colizzi M, Weltens N, McGuire P, Van Oudenhove L and Bhattacharyya S (2019b) Descriptive psychopathology of the acute effects of intravenous delta-9-tetrahydrocannabinol administration in humans. *Brain Sciences* 9(4), 93. 10.3390/brainsci9040093 PMC6523579 31027219

[ref12] Dhunjibhoy JE (1930) A brief resume of the types of insanity commonly met with in India with a full description of “Indian hemp insanity” peculiar to the country. *Journal of Mental Science* 76(313), 254–264.

[ref13] Di Forti M, Marconi A, Carra E, Fraietta S, Trotta A, Bonomo M, Bianconi F, Gardner-Sood P, O’Connor J, Russo M, Stilo SA, Marques TR, Mondelli V, Dazzan P, Pariante C, David AS, Gaughran F, Atakan Z, Iyegbe C, Powell J, Morgan C, Lynskey M and Murray RM (2015) Proportion of patients in south London with first-episode psychosis attributable to use of high potency cannabis: A case-control study. *Lancet Psychiatry* 2(3), 233–238. 26359901 10.1016/S2215-0366(14)00117-5

[ref14] Di Forti M, Quattrone D, Freeman TP, Tripoli G, Gayer-Anderson C, Quigley H, Rodriguez V, Jongsma HE, Ferraro L, La Cascia C, La Barbera D, Tarricone I, Berardi D, Szöke A, Arango C, Tortelli A, Velthorst E, Bernardo M, Del-Ben CM, Menezes PR, Selten JP, Jones PB, Kirkbride JB, Rutten BP, de Haan L, Sham PC, van Os J, Lewis CM, Lynskey M, Morgan C, Murray RM **and** EU-GEI WP2 Group (2019) The contribution of cannabis use to variation in the incidence of psychotic disorder across Europe (EU-GEI): A multicentre case-control study. *Lancet Psychiatry* 6(5), 427–436. 30902669 10.1016/S2215-0366(19)30048-3 PMC7646282

[ref15] Dugas AF, Jalalpour M, Gel Y, Levin S, Torcaso F, Igusa T and Rothman RE (2013) Influenza forecasting with Google Flu Trends. *PLoS One* 8(2), e56176. 10.1371/journal.pone.0056176 PMC3572967 23457520

[ref16] Ferdinand RF, Sondeijker F, van der Ende J, Selten JP, Huizink A and Verhulst FC (2005) Cannabis use predicts future psychotic symptoms, and vice versa. *Addiction* 100(5), 612–618. 15847618 10.1111/j.1360-0443.2005.01070.x

[ref17] Flanagan R, Kuo B and Staller K (2021) Utilizing Google Trends to assess worldwide interest in irritable bowel syndrome and commonly associated treatments. *Digestive Diseases and Sciences* 66(3), 814–822. 32361922 10.1007/s10620-020-06290-7

[ref18] Frisher M, Crome I, Martino O and Croft P (2009) Assessing the impact of cannabis use on trends in diagnosed schizophrenia in the United Kingdom from 1996 to 2005. *Schizophrenia Research* 113(2-3), 123–128. 19560900 10.1016/j.schres.2009.05.031

[ref19] Henquet C, Krabbendam L, Spauwen J, Kaplan C, Lieb R, Wittchen HU and van Os J (2005) Prospective cohort study of cannabis use, predisposition for psychosis, and psychotic symptoms in young people. *BMJ* 330(7481), 11. 10.1136/bmj.38267.664086.63 PMC539839 15574485

[ref20] Johnson AK and Mehta SD (2014) A comparison of Internet search trends and sexually transmitted infection rates using Google Trends. *Sexually Transmitted Diseases* 41(1), 61–63. 24326584 10.1097/OLQ.0000000000000065

[ref21] Marconi A, Di Forti M, Lewis CM, Murray RM and Vassos E (2016) Meta-analysis of the association between the level of cannabis use and risk of psychosis. *Schizophrenia Bulletin* 42(5), 1262–1269. 26884547 10.1093/schbul/sbw003 PMC4988731

[ref22] Mathieu E, Ritchie H, Ortiz-Ospina E, Roser M, Hasell J, Appel C, Giattino C and Rodés-Guirao L (2021) A global database of COVID-19 vaccinations. *Nature Human Behavior* 5(7), 947–953. 10.1038/s41562-021-01122-8 33972767

[ref23] McGrath J, Saha S, Welham J, El Saadi O, MacCauley C and Chant D (2004) A systematic review of the incidence of schizophrenia: The distribution of rates and the influence of sex, urbanicity, migrant status and methodology. *BMC Medicine* 2, 13. 10.1186/1741-7015-2-13 PMC421751 15115547

[ref24] Moore TH, Zammit S, Lingford-Hughes A, Barnes TR, Jones PB, Burke M and Lewis G (2007) Cannabis use and risk of psychotic or affective mental health outcomes: A systematic review. *Lancet* 370(9584), 319–328. 17662880 10.1016/S0140-6736(07)61162-3

[ref25] Moreau JJ (1845) *Du hachisch et de l’aliénation mentale: études psychologiques*. Paris: Fortin Masson.

[ref26] Murray RM, Englund A, Abi-Dargham A, Lewis DA, Di Forti M, Davies C, Sherif M, McGuire P and D’Souza DC (2017) Cannabis-associated psychosis: Neural substrate and clinical impact. *Neuropharmacology* 124, 89–104. 28634109 10.1016/j.neuropharm.2017.06.018

[ref27] Niemi-Pynttari JA, Sund R, Putkonen H, Vorma H, Wahlbeck K and Pirkola SP (2013) Substance-induced psychoses converting into schizophrenia: A register-based study of 18,478 Finnish inpatient cases. *Journal of Clinical Psychiatry* 74(1), e94–e99. 23419236 10.4088/JCP.12m07822

[ref28] Nuti SV, Wayda B, Ranasinghe I, Wang S, Dreyer RP, Chen SI and Murugiah K (2014) The use of Google Trends in health care research: A systematic review. *PLoS One* 9(10), e109583. 10.1371/journal.pone.0109583 PMC4215636 25337815

[ref29] Power RA, Verweij KJ, Zuhair M, Montgomery GW, Henders AK, Heath AC, Madden PA, Medland SE, Wray NR and Martin NG (2014) Genetic predisposition to schizophrenia associated with increased use of cannabis. *Molecular Psychiatry* 19(11), 1201–1204. 24957864 10.1038/mp.2014.51 PMC4382963

[ref30] Prasanth S, Singh U, Kumar A, Tikkiwal VA and Chong PHJ (2021) Forecasting spread of COVID-19 using Google Trends: A hybrid GWO-deep learning approach. *Chaos, Solitons, and Fractals* 142, 110336. 10.1016/j.chaos.2020.110336 PMC7580652 33110297

[ref31] Schoeler T, Petros N, Di Forti M, Klamerus E, Foglia E, Ajnakina O, Gayer-Anderson C, Colizzi M, Quattrone D, Behlke I, Shetty S, McGuire P, David AS, Murray R and Bhattacharyya S (2016) Effects of continuation, frequency, and type of cannabis use on relapse in the first 2 years after onset of psychosis: An observational study. *Lancet Psychiatry* 3(10), 947–953. 27567467 10.1016/S2215-0366(16)30188-2

[ref32] Schootman M, Toor A, Cavazos-Rehg P, Jeffe DB, McQueen A, Eberth J and Davidson NO (2015) The utility of Google Trends data to examine interest in cancer screening. *BMJ Open* 5(6), e006678. 10.1136/bmjopen-2014-006678 PMC4466617 26056120

[ref33] Spalletta G, Bria P and Caltagirone C (2007) Differences in temperament, character and psychopathology among subjects with different patterns of cannabis use. *Psychopathology* 40(1), 29–34. 17057422 10.1159/000096387

[ref34] Vasconcellos-Silva PR, Carvalho DBF, Trajano V, de La Rocque LR, Sawada A and Juvanhol LL (2017) Using Google Trends data to study public interest in breast cancer screening in Brazil: Why not a pink February? *JMIR Public Health and Surveillance* 3(2), e17. 10.2196/publichealth.7015 PMC5399222 28385679

